# Transperitoneal laparoscopic live donor nephrectomy: Current status

**DOI:** 10.4103/0970-1591.33727

**Published:** 2007

**Authors:** A. Srivastava, N. Gupta, Anant Kumar, Rakesh Kapoor, Deepak Dubey

**Affiliations:** Department of Urology and Renal Transplantation, SGPGIMS, Lucknow, India

**Keywords:** Donor, laparoscopy, recipient, transperitoneal, transplant

## Abstract

Renal transplantation is the treatment of choice for a suitable patient with end stage renal disease. Unfortunately, the supply of donor organs is greatly exceeded by demand. In many countries the use of kidneys from living donors has been widely adopted as a partial solution. Traditionally donor nephrectomy has been performed via a open flank incision however with some morbidity like pain and a loin scar. Currently, the donor nephrectomy is increasingly being performed laparoscopically with the objective of reducing the morbidity. It is also hoped that this will lead to increasing acceptance of living donation. The first minimally invasive living donor nephrectomy was carried out in 1995 at the Johns Hopkins Medical Center and since then many centers have undertaken laparoscopic living donor nephrectomy. The laparoscopic approach substantially reduces the donor morbidity and wound related problems associated with open nephrectomy. The laparoscopic techniques thus have the potential to increase the number of living kidney donors. The present article attempts to review the safety and efficacy of transperitoneal laparoscopic donor nephrectomy.

End stage renal disease (ESRD) patients have the option of lifelong maintenance hemodialysis, continuous ambulatory peritoneal dialysis (CAPD) or kidney transplantation and of these, kidney transplantation is the treatment of choice for a suitable patient.[1] Unfortunately, the supply of donor organs is greatly exceeded by demand. In many countries the use of kidneys from living donors has been widely adopted as a partial solution.[2] Donor nephrectomy via an open and usually flank incision is relatively safe. However, some morbidity is associated with open donor nephrectomy which may deter potential living donors from volunteering and candidate transplant recipients from requesting a living donor. Recently, donor nephrectomy has been performed using minimally invasive surgery. The first laparoscopic living donor nephrectomy was carried out in 1995 at the Johns Hopkins Medical Center and since then many centers have undertaken laparoscopic living donor nephrectomy. The laparoscopic approach substantially reduces the donor morbidity and wound-related problems associated with open nephrectomy.[3–5] The laparoscopic techniques thus have the potential to increase the number of living kidney donors.[6,7] The present article attempts to review the safety and efficacy of transperitoneal lap donor nephrectomy.

## STANDARD OPERATIVE TECHNIQUE

Laparoscopic live donor nephrectomy (LLDN) can be done transperitoneally or retroperitoneally on either side. The operative technique of LLDN has undergone many refinements since it was first described. The approach is most commonly transperitoneal, which allows adequate working space and easy dissection.

## LEFT TRANSPERITONEAL APPROACH

The donor is placed in a modified lateral decubitus position. Pneumoperitoneum is created by insufflation of carbon dioxide using a veress needle or after introduction of a laparoscopic 12 mm port at the umbilicus by open Hassan technique. The umbilical port is primarily used as a camera port. Another 12 mm laparoscopic port is placed between the umbilical port and anterior superior iliac spine (spinoumbilical port). A 5 mm port is placed in line with the camera port about 3c A fourth 5 mm laparoscopic port may be used for retraction, if needed and is placed 4 cm below the costal margin in the anterior axillary line.[8]

The lateral peritoneal reflection is incised along the line of Toldt from the splenic flexure to the pelvic inlet and dissection is continued in the plane between Gerota's fascia and descending colonic mesentry. The left gonadal vein is followed up to the left renal vein. Before beginning the hilar dissection 25 mg of mannitol is administered. At least 3–4L of fluid is given during surgery to negate the effect of pneumoperitoneum.[9] The adrenal and lumbar veins are clipped and divided and the renal vein is mobilized. The left renal artery is dissected at its aortic origin. If vasospasm is noted, the renal artery can be bathed in a papaverine solution (30 mg/ml). The adrenal gland is separated from the upper pole of the kidney. After completing the hilar dissection but before ligating the vessels, the patient is given another 25 mg of mannitol and some centers also give 20 mg of frusemide. Vigorous intravenous hydration, mannitol/frusemide and topical papaverine instillation on the renal artery help to minimize pneumoperitoneum pressure-induced oliguria.[10,11] The gonadal vein is usually not divided near the renal vein, instead it is lifted up and using this as a guide, the dissection is continued on its medial border to prevent the compromise of the periureteric blood supply. No dissection is done between the gonadal vein and ureter. The ureteral packet with generous periureteric fatty tissue is dissected up to the level of iliac vessels and the gonadal vein is clipped and divided where it crosses over the ureter. The lower pole of the kidney is elevated and the remaining posterior attachments divided with sharp and blunt dissection. The ureter is clipped distally and divided immediately above the level of the iliac vessels, leaving its proximal end open for the remainder of the dissection.

By placing forceps between previously mobilized renal artery and vein, excellent exposure of renal hilum is possible. The renal artery and vein are sequentially ligated with a GI vascular stapler or 13 mm Weck Clips (Hemolock, Weck Pilling and USA). The kidney is then carefully extracted from the pfannenstiel incision.

## RIGHT TRANSPERITONEAL APPROACH

The transperitoneal laparoscopic approach may be similar to that on the left side. Modifications have been made in right LLDN to compensate for shorter vessels and to prevent thrombotic complications. In the recipient, the iliac vein is completely mobilized by dividing all of its posterior branches to facilitate making a tension-free anastomosis.[12] The dissection of the interaortocaval space to allow the division of the renal artery at its origin from the aorta has been recommended by some investigators.[13] This maneuver allows for the division of the right renal artery at its origin, rather than at the lateral border of the vena cava. Another technique to gain arterial length involves mobilizing the vena cava by dividing the lumbar veins.[14] This allows the vena cava to be rolled anteriorly and the kidney to be placed in a “flipped” position, increasing exposure of the right renal artery at its origin from the aorta. The kidney is then retracted laterally and endovascular stapling device is placed parallel to the aorta at the origin of the right renal artery, thus allowing for the maximization of arterial length.

Insertion of an Endo-GIA stapler through the port positioned in the right lower abdominal quadrant allows control of the renal vein at its junction with the vena cava, preserving the maximum possible length of renal vein.[15] To maximize the length of the right renal vein, one of three modifications is done.[12] The first involves use of a TA stapler which fires two staple lines without cutting. The vein is subsequently cut flush with the staple line to gain extra length. The stapler is introduced through the lateral trocar site, in a plane parallel to the inferior vena cava. The parallel orientation of the stapler in relation to the vena cava results in preservation of additional right renal vein length.[16] The second modification involves open surgical division of the right renal vein. A 5-6 cm right subcostal incision is made. After laparoscopic dissection of the right kidney, a Satinsky clamp is placed on the inferior vena cava just medial to the origin of the right renal vein. Alternatively, a laparoscopic Satinsky clamp can be used to obtain a cuff of the vena cava, which is then repaired intracorporeally.[17] A third modification is used in case of a short renal vein and a graft of the recipient's saphenous vein can be used to reconstruct the renal vein.

However, with increasing experience more and more donors can be taken up for left transperitoneal approach as multiple vessels and other vascular anomalies are no more a contraindication for this procedure.

## RESULTS IN TERMS OF DONOR OUTCOME

At many centers, the number of individuals willing to undergo LLDN today would not have donated a kidney if open surgery was the only option available for organ donation. This is so because LLDN offers decreased postoperative pain, marked cosmetic benefit and better convalescence compared to open donor nephrectomy.

In most reported experiences, the operating time at the beginning of the LDN learning curve was generally longer than for open live donor nephrectomy.[18] In a meta-analysis of published reports regarding the current status of LDN, the operating time was significantly longer (183 to 340 versus 95 to 260 min, *P* < 0.05) in LDN series compared with open donor nephrectomy series.[19] However, with increasing experience it decreases and tends to plateau after about 25 cases.[20] Our mean operative time for transperitoneal LDN is 180 min and sometimes we have finished the operation in 120 min [Table 1].

**Table 1:** Experience with transperitoneal laparoscopic live donor nephrectomy

	No. of patients	Warm ischemia time (min)	EBL (ml)	OR time (min)	Open conversion	Hosp. stay (days)
LDN	342	4.5	85	180	12	3.14
ODN	1000	2	220	110	.	5.7

Laparoscopic live donor nephrectomy and open donor nephrectomy are comparable in the estimated blood loss and postoperative transfusion requirement.[21–23] In fact most series reported lesser blood loss in the laparoscopic group compared to the open group [Table 2].

**Table 2:** Complications

Hemorrhage requiring blood transfusion	22
Bowel injury	2
Reoperation	4
Wound infection	22
Pneumonitis	7
Hydrothorax	1
Excessive drain output	2
Unexplained anemia	3
Ureteral obstruction	1
Incisional hernia	2
Pseudohernia	1
Accessory renal artery thrombosis	0

The reported frequency of open conversion during LDN ranges from 0 to 13%.[23] The most common causes of conversion to open donor nephrectomy are intraoperative hemorrhage or vascular injury (65%), difficult kidney exposure or obese donor (20%), vascular stapler malfunction (12%) and pneumoperitoneum loss (3%).[19] Sometimes it is difficult to proceed due to dense adhesions around the renal hilum and prominent lymphatics [Table 3].

**Table 3:** Conversions

Total conversions	12
Bleeding	6
Failure to proceed	4
Bowel injury	2

Reduced postoperative pain and recuperative time, the major advantages of LDN, have been demonstrated in several studies. Ratner *et al.*[24] concluded that the amount of parenteral analgesia given to donors after LDN was significantly lower than after open donor nephrectomy (*P* < 0.05). Similar results have been found in other series[25] [Table 4].

**Table 4:** Donor outcome

	ODN (n = 1000)	LLDN (n = 342)
PO intake (days)	4.5	2.4
Hospital stay (days)	5.7	3.14
Analgesic usage (mg)	251	150
Driving (wks)	5.3	3.0
Caring for home (wks)	6.2	2.5
Full activity (wks)	7.2	4.4
Return to work (wks)	12	8
Exercising (wks)	6.4	3.6

Comparing LDN to open living donor nephrectomy (OLDN), the hospital stay, resumption of oral intake, the time to return home have been found to be significantly favorable to the LDN group[23,26,27] [Table 4].

Several series have addressed donor quality of life following LDN and ODN. It has been shown that LDN resulted in a shorter time until patients were able to drive, take care of the home and return to full activity, work and regular exercise.[26,27] In the comparison of pure laparoscopic live donor nephrectomy versus hand-assisted laparoscopic live donor nephrectomy, the former showed a shorter time to return to normal physical activity and work[28] [Table 4].

## RECIPIENT'S OUTCOME

The warm ischemia time (WIT) presents a major concern as it has always been slightly longer in the laparoscopic group when compared with open donor nephrectomy due to the longer extraction time. It has been thought that any increase in this would translate into a poor graft function. This notion has been disproved by various studies suggesting no bearing of this small difference on the recipient outcome. The warm ischemia time during LDN may range between 95 and 300 seconds.[20,21] At the University of Maryland, in 738 cases performed during a six-year period, the warm ischemia time was 169 ± 90.8 seconds.[29] The warm ischemia time can be reduced with increasing experience, as shown by Rawlins *et al*. (3.3 min in the first 25 LDN cases versus 1.8 min in the most recent 25 LDNs (*P*< 0.001).[20] A similar result has been shown by Soulsby *et al*.[11] The effects of WIT on delayed graft function was assessed extensively by Jacob *et al*., where the rate of decline in serum creatinine concentration, S Cr. in the first 10 days, changes in S Cr. at three months, acute rejection rate, biopsy-proven chronic allograft rejection and graft survival were assessed according to the duration of WIT.[29] Analysis was done by comparing WIT ≤ 3 versus ≥ 3 min and WIT ≤ 5, 5-10 and ≥ 10 min. Prolonged WIT did not appear to have an effect on early graft function or the rate of decline in serum creatinine during the first three months post-transplantation.

We evaluated the impact of warm ischemia time in LDN. It did not correlate with the incidence of delayed graft function, acute rejection or allograft or recipient survival [Table 5].

**Table 5:** Recipient outcome

	ODN n=1000	LDN n=342	*P* value
S. Creat. (preop.)	5.1	5.34	ns
S. Creat. (Day 1)	2.36	2.56	ns
S. Creat. (Day 3)	1.71	1.63	ns
S. Creat. (Day 7)	1.69	1.72	ns
S. Creat. (1M)	1.25	1.28	ns
S. Creat. (3M)	1.35	1.42	ns
S. Creat. (6 M)	1.46	1.41	ns
S. Creat. (1year)	1.51	1.52	ns
Return to normal S. Creat.(Day 2) %	56%	51%	ns
Return to normal S. Creat.(Day 5)%	78%	76%	ns
Rejection (3 M) %	27.5%	25.4%	ns
ATN (3 M) %	12.9%	13.5%	ns

Several clinical trials have shown no significant difference in the serum creatinine level between open and LDN at three days, 30 days and three months after transplantation.[30] The longer warm ischemia time in the laparoscopic group has been shown not to affect the long-term graft outcome. In a systematic review of 24 comparative studies for both ODN and LDN, the trend was for values to start at approximately 4.0 to 5.0 mg/dl on POD1 but to drop to approximately 1.5 by POD7 and to stabilize at approximately that level thereafter.[24] At a follow-up of six months, Rawlins *et al*. confirmed no significant difference in serum creatinine level in LDN versus open donor nephrectomy group (1.64 versus 1.48 mg/dL, respectively, *P* =0.26).[20]

In a comparison between open donor nephrectomy and LDN in patients with multiple renal arteries, the postoperative serum creatinine level after one year of follow-up has been found to be similar (1.3 mg/dL versus 1.3 mg/dL, respectively, *P* = 0.9) and the graft survival rate (87.5% versus 87.5%, respectively) has also been similar.[24] Regardless of the procurement technique (open versus laparoscopic), live donor kidney transplantation was associated with a delayed graft function rate of 5-10%.[25]

An analysis of risk factors potentially affecting delayed graft function after LLDN had shown that recipient age, donor/recipient sex relationship, unrelated highly mismatched donors and cold/total preservation time to be associated with impaired renal function recovery. However, the laparoscopic approach was not related to delayed graft function[19,30] [Table 5].

There have been concerns about vessel and ureteral length which could be harvested in LDN as it would have a direct bearing on the recipient operation. In a randomized controlled trial[18] studying the structural and functional aspects of LDN and ODN it was shown that the left renal vein (*P*= 0.14) and left renal artery length (*P* = 0.38), right renal vein (*P*= 0.38) and artery length (*P*= 0.33) were similar. Ureteric length was significantly greater in the LDN group for both left and right nephrectomy. This study demonstrated that LDN yielded kidneys that were structurally and functionally equivalent to those acquired by the open operation.

In initial LDN series, ureteral injuries occurred more frequently than during open donor nephrectomy (0-11% versus 0-6%, respectively).[19] Subsequent technical modifications (e.g. preservation of the peri ureteral tissue allowing adequate ureteral blood supply) have reduced the incidence of such complications. Refinements in surgical technique allowed Ratner *et al*. to reduce an initial 9.1% incidence of ureteral complications in the first 110 cases to 3% in the last 100 cases.[7] The University of Maryland has reported ureteral complications initially, about 7%, which have gradually decreased to about 2.5%[29] [Table 2].

A systematic review of reported LDN series found no significant differences between the rate of acute rejection after LDN (2–30%) and open donor nephrectomy (0–32%).[19] Similar results have been reported from the University of Maryland with more than 700 cases wherein early graft rejection occurred in 3% of cases[29] [Table 5].

## COST ANALYSIS

Wolf *et al*. reported a higher operating cost in LDN series (73% greater) compared with open donor nephrectomy.[21] However, when we consider the total cost of the procedure including hospital stay, analgesic requirement, loss of days of work and the need for supportive care LDN fares better compared to ODN not adding the additional benefit of better cosmesis.[31] Presently at our institute ODN costs US$ 350 vs. LDN US$ 500. This constitutes the total cost incurred during stay in the hospital.

## CONCLUSION

Laparoscopic donor nephrectomy has now been established as a safe and efficacious procedure, especially with refinements in equipment and surgical techniques. It provides all the benefits of minimal invasive surgery to the most unique patient that is the donor while maintaining results equivalent to the ODN in terms of graft function and recipient outcome. Making modifications in the original procedure can significantly bring down the cost, thus making it a procedure of choice for developing nations.
